# Stem Cells From Human Exfoliated Deciduous Teeth-Conditioned Medium (SHED-CM) is a Promising Treatment for Amyotrophic Lateral Sclerosis

**DOI:** 10.3389/fphar.2022.805379

**Published:** 2022-02-03

**Authors:** Tomoyuki Ueda, Taisei Ito, Masatoshi Inden, Hisaka Kurita, Akihito Yamamoto, Isao Hozumi

**Affiliations:** ^1^ Laboratory of Medical Therapeutics and Molecular Therapeutics, Gifu Pharmaceutical University, Gifu, Japan; ^2^ Department of Tissue Regeneration, Institute of Biomedical Sciences, Tokushima University, Tokushima, Japan

**Keywords:** amyotrophic lateral sclerosis, copper-zinc superoxide dismutase 1, stem cells from human exfoliated deciduous teeth, induced pluripotent stem cells, aggregation

## Abstract

Amyotrophic lateral sclerosis (ALS) is a neurodegenerative disorder, characterized by the loss of upper and lower motor neurons, for which an effective treatment has yet to be developed. Previous reports have shown that excessive oxidative stress, related to mitochondrial dysfunction and the accumulation of misfolding protein, contributes to ALS pathology. In terms of treatment, it remains necessary to identify effective medicines for multiple therapeutic targets and have additive effects against several disorders. In this study, we investigated stem cells from human exfoliated deciduous teeth (SHED), which release many factors, such as neurotrophic factors and cytokines, and are applied to treat neurological diseases. Specifically, we examined whether SHED-conditioned medium (CM), i.e., the serum-free culture supernatant of SHED, reduced mutant SOD1-induced intracellular aggregates and neurotoxicity. We found that SHED-CM significantly suppressed the mutant SOD1-induced intracellular aggregates and neurotoxicity. The neuroprotective effects of SHED-CM are partly related to heat shock protein and the activation of insulin-like growth factor-1 receptor. SHED-CM also had a protective effect on induced pluripotent stem cell-derived motor neurons. Moreover, SHED-CM was effective against not only familial ALS but also sporadic ALS. Overall, these results suggest that SHED-CM could be a promising treatment for slowing the progression of ALS.

## Introduction

Amyotrophic lateral sclerosis (ALS) is a severe intractable neurodegenerative disease characterized by the degeneration of upper and lower motor neurons. The initial symptoms of ALS are weakness of the muscles in the hands, feet, tongue, and throat, as well as bulbar symptoms such as dysphagia and dyspnea; eventually, ALS leads to death due to respiratory muscle paralysis (Julien et al., 2001; Ling, et al., 2013; Norris et al., 2020).

Significant geographical differences exist in the clinical features of ALS. In Europe and the USA, the incidence of ALS is estimated at 1.75–3.00 cases per 100,000 people per year and the prevalence is 10–12 cases per 100,000 people. Men have a higher risk of sporadic ALS than women, with a global male-to-female ratio of 1.2–1.5 (Julien et al., 2001; Mathis, et al., 2019). About 5%–10% of ALS patients suffer from hereditary ALS, whereas about 90%–95% suffer the onset of ALS regardless of genetics. In the last 20 years, chromosome 9 open reading frame 72 (C9ORF72), Cu/Zn superoxide dismutase (SOD1), TAR DNA-binding protein 43 (TARDBP), and fused in sarcoma/translated in liposarcoma (FUS) have been revealed as the most commonly mutated genes in ALS (Julien et al., 2001; Mathis, et al., 2019).

In particular mutations of SOD1, first discovered as an ALS-associated gene in 1993, account for about 20% of familial ALS (FALS) (also known as ALS1). Several studies have shown oxidative stress as a feature of ALS pathogenesis (Liu, et al., 2015; Bond, et al., 2018), which involves increased production of reactive oxygen species (ROS) and oxidative damage to proteins and lipids (D’Amico, et al., 2013). In addition, excessive endoplasmic reticulum (ER) stress, mitochondrial dysfunction, and disruption of the degradative system (e.g., autophagy, ubiquitin proteasome, etc.) have been associated with the progression and development of ALS. Furthermore, these disorders cause the accumulation of abnormal aggregates in motor neurons (Kikuchi et al., 2006; Cheroni, et al., 2009; Shi et al., 2010). Evidence now indicates that suppressing the accumulation of aggregates is the most effective therapy against ALS.

Several mutant SOD1 mouse models have been created that present mitochondrial dysfunction, aggregate accumulation of SOD1, and neuronal cell death; effective therapeutic agents against ALS have been identified using such models (Gurney et al., 1994; Bruijn, et al., 1997). However, effective drugs for treating patients with ALS have yet to be developed. Although two therapeutic medicines for ALS treatment are on the market and are in clinical use, riluzole (a potential antiglutamatergic agent) and edaravone (a free-radical scavenger) (Yoshino et al., 2019), these medicines only prolong patient survival by a few months. Furthermore, these two agents are not necessarily effective when used in ALS model mice. Thus, identifying novel therapeutic agents for ALS is challenging. Indeed, it is difficult to suppress the onset and progression of the disease with a single drug; it is more important to suppress ALS with a combination of agents. This strategy applies not only to ALS but also to other neurodegenerative diseases such as Alzheimer’s disease (Kondo, et al., 2017). Therefore, there is a need to identify effective drugs for multiple therapeutic targets in ALS and drugs that have additive effects against the disease.

Accordingly, we investigated stem cells from human exfoliated deciduous teeth (SHED), which have become a focus in the field of regenerative medicine. SHED, which are localized around blood vessels in the pulp of deciduous teeth, were isolated from deciduous teeth for the first time in 2003. They are an ideal stem cell source and have great potential in stem cell therapy. In addition, SHED can differentiate into various cells such as chondrocytes, endothelial cells, and functionally and structurally active neurons (Miura, et al., 2003; Fujii, et al., 2015). Previous reports showed that SHED release many factors, such as neurotrophic factors, and promote recovery from central neuropathy (Inoue, et al., 2013; Yamagata, et al., 2013). Furthermore, SHED-conditioned medium (CM), which contains a variety of growth factors such as insulin and hepatocyte growth factor (HGF) at levels much higher than those found in bone marrow-derived stem cell-CM (Matsubara, et al., 2015), is expected to have protective effects. Recently, preclinical studies using animal models have shown the therapeutic abilities of SHED. Indeed, SHED-CM has protective effects against Alzheimer’s disease, acute lung injury, and diabetes (Izumoto-Akita, et al., 2015; Mita, et al., 2015; Wakayama, et al., 2015). In ALS model mice, dental pulp stem cell-derived serum-free culture supernatant has been reported to suppress the loss of motor function (Wang, et al., 2019). However, the effects of SHED-CM against ALS have not yet been revealed.

In the present study, we investigated whether SHED-CM has neuroprotective effects against mutant SOD1-induced neurotoxicity in a cellular model and in patient-derived disease-specific induced pluripotent stem (iPS) cells. Additionally, we investigated whether SHED-CM reduces intracellular aggregates of mutant SOD1. This research could lead to analysis of the additive effects of various active factors released from SHED and the development of novel therapeutic agents for ALS.

## Manuscript Formatting

### Materials and Methods

#### Ethics

All experiments on iPS cells and SHED-CM were approved by the Ethics Committees of Gifu Pharmaceutical University and Gifu University, and they were performed in accordance with the Ethical Guidelines for Medical and Health Research Involving Human Subjects in Japan as well as the Ethical Guidelines for Human Genome/Gene Analysis Research in Japan (Gifu Pharmaceutical University approval numbers: 1–23 and 1–25; Gifu University approval numbers: 29–501 and 2020–162; registered ID: UMIN000038561and 000030101).

#### Plasmid Cell Culture and Transfection

As in our previous study, we used expression plasmids (pmCherry-N1; Clontech Laboratories Inc., CA, USA) harboring human SOD1 (wild-type, G85R, or G93A) (Ueda et al., 2018; Ueda et al., 2019). Briefly, N2a cells were maintained in Dulbecco’s modified Eagle medium (DMEM; Wako Pure Chemical Industries, Ltd., Osaka Japan) containing 10% (v/v) fetal bovine serum (FBS; Thermo Fisher Scientific Inc., Waltham, MA, USA). The cells were passaged by trypsinization every 3–4 days. Transient plasmid expression in N2a cells was accomplished with Lipofectamine 2000 according to the manufacturer’s protocol (Thermo Fisher Scientific Inc.).

#### Cell Viability and Neurotoxicity Assays

Cell viability and neurotoxicity assays were performed according to the methods in our previous study (Ueda, et al., 2018; Ueda, et al., 2019). Briefly, for cell viability, N2a cells were seeded at 1.0 × 10^5^ cells/ml in 96-well plates in DMEM containing 10% FBS. Following plasmid transfection, the cells were differentiated for 48 h in low glucose (1.0 g/L glucose) DMEM supplemented with 2% FBS and 2-mM N,N-dibutyladenosine 3′,5′-phosphoric acid (dbcAMP; Nacalai Tesque, Kyoto, Japan) N2a cells expressing mCherry-SOD1^G85R^ incubated for 48 h with SHED-CM and neutralizing IGF-2 antibodies (bio-techne), Insulin-like Growth Factor-II, Human, recombinant (rhIGF-II: 0.5 ng/ml, 48 h) (Wako Pure Chemical Industries Ltd.) and a HSP 70 inhibitor, Pifithrin mu (Pi mu: 1 µM, 48 h) (StressMarq Biosciences Inc.). The number of live cells was estimated using a Cell Counting Kit-8 (Wako Pure Chemical Industries Ltd.). Briefly, the reagent was added to the wells and the plate was incubated at 37°C for 2 h. Cell viability was calculated by detecting the optical density of formazan at 450 nm using GloMax^®^ (Promega, Madison, WI, USA). A 600-nm wavelength was used as a reference. For neurotoxicity, N2a cells were also seeded at 1.0 × 10^5^ cells/ml in 96-well plates in DMEM containing 10% FBS. Following plasmid transfection, the cells were differentiated for 48 h in low glucose (1.0 g/L glucose) DMEM or SHED-CM supplemented with 2% FBS and 2-mM dbcAMP. The number of dead cells was estimated using an LDH Assay Kit (Wako Pure Chemical Industries Ltd.). Briefly, the reagent was added to the wells and the plate was incubated at room temperature for 30 min. Cell toxicity was calculated by detecting the optical density of formazan at 490 nm using GloMax^®^. In this case, a 630-nm wavelength was used as a reference.

#### Aggregation Rate Analysis

After 24 h of vector transfection into N2a cells, the cells were treated with SHED-CM for 24 h and neutralizing IGF-2 antibodies (bio-techne), Insulin-like Growth Factor-II, Human, recombinant (rhIGF-II: 0.5 ng/ml, 24 h) (Wako Pure Chemical Industries Ltd.) and Pifithrin mu (Pi mu: 1 µM, 24 h) (StressMarq Biosciences Inc.). Then subsequently washed twice with PBS for 5 min before being fixed with 4% paraformaldehyde for 15 min. Fluorescent microscopy images were acquired with a confocal fluorescence microscope (LSM700; Carl Zeiss). To count the number of aggregates, we used an IN Cell Investigator 2200 (GE Healthcare, Buckinghamshire, UK). In each experiment, at least 3,000 cells were counted. To quantify aggregate formation, the intensity of even and diffuse fluorescence was set as a threshold and the number of cells showing over-threshold fluorescence intensities within each field was counted. In particular, three or more aggregates in a single cell were counted as intracellular aggregate cells. To measure the percentage of SOD1 aggregates. First, we stained nuclei with Hoechst 33342 and measured the number of all cells. After that, we measured the number of cells in which SOD1 aggregation and then, the percentage (mCherry positive cells/Hoechst positive cells) was calculated in the study. Following threshold analysis, the presence of aggregates within each field was confirmed by eye, based on methods from previous studies (Ueda et al., 2018; Ueda et al., 2019).

#### Western Blot Analysis

Western blot analysis was completed according to the method from our previous study (Ueda et al., 2019; Ueda et al., 2018). Briefly, after 24 h of vector transfection into N2a cells, the cells were treated with SHED-CM (30%, 50%, 70%/24 h). The cells were then lysed with RIPA buffer [50-mM Tris-HCl (pH. 7.4), 150-mM NaCl, 0.1% Triton-X, 0.5% sodium deoxycholate, 0.1% sodium dodecyl sulfate (SDS), protease inhibitor cocktail, and phosphatase inhibitor] before being centrifuged at 15,000 × g and 4°C for 30 min. Protein concentration was measured using a BCA Protein Assay Kit (Thermo Fisher Scientific Inc.) with bovine serum albumin (BSA) as the standard. Lysates were mixed with sample buffer containing 10% 2-mercaptoethanol and subjected to 12%, 10%, and 8% SDS-polyacrylamide gel electrophoresis (SDS-PAGE). To separate proteins of a given molecular weight, 20 µg of protein were applied to SDS-PAGE. SDS-PAGE was performed under a constant 200 V at room temperature for 40 min. The proteins that were separated into poly acrylamide gel were transferred to a PVDF membrane in transfer buffer (0.3% Tris, 1.44% glycine, and 20% methanol) under a constant 100 V at 4°C for 90 min. First, the membranes were incubated for 60 min with 5% BSA (Wako) and then incubated overnight with the following primary antibodies: mouse monoclonal antibodies against Bip, Chop (1:1,000; Cell Signaling Technology, Danvers, USA), and β-actin (1: 2,000; Santa Cruz Biotechnology, Dallas, TX USA), and rabbit antibodies HSP90, HSP70, HSP60, pIGF-1receptor, p-AKT, AKT, p-ERK, ERK, p-GSK-3β, and GSK-3β (1:1,000; Cell Signaling Technology). Following the reaction with primary antibodies, the PVDF membrane was washed twice with TBS-T for 7 min. Then the membranes were incubated with secondary antibodies [goat anti-rabbit antibody conjugated with HRP (1:2,500; Millipore) and goat anti-mouse HRP antibody conjugated with HRP (1:2,500; Millipore)]. Following the reaction with secondary antibodies, the PVDF membrane was washed twice with TBS-T for 7 min. Finally, they were incubated in ECL prime (GE Healthcare) to generate HRP antibody chemiluminescence, which was detected using a Fusion system (Vilber-Lourmat). Image J software (NIH, New York, NY, USA) was used to measure band density.

#### Immunoprecipitation

Cells were lysed with RIPA buffer (contents detailed in section 3.1.5.) and then centrifuged at 15,000 × g and 4°C for 30 min. The immunoprecipitation procedure was performed using Protein G magnetic beads (Millipore). Protein G was incubated until the supernatant became bound to antimisfolded SOD1 antibody (MS785/MS27) at 4°C overnight. The next day, beads were collected by magnets and washed three times. Lysates were mixed with sample buffer containing 10% 2-mercaptoethanol and subjected to 12% SDS-PAGE. Membranes were blocked with Blocking One (Nacalai Tesque) and immunoblotted with SOD1 antibodies (1:1,000; ENZO). Finally, they were incubated in ECL prime (GE Healthcare) to generate HRP antibody chemiluminescence, which was detected using the aforementioned Fusion system.

#### ROS Production

ROS production about oxidative stress was measured according to methods reported in our previous study (Ueda T et al., 2019; Ueda T et al., 2018). Briefly, after 24 h of vector transfection into N2a cells, the cells were treated with SHED-CM (30%, 50%, 70%/24 h). To detect mutant SOD1-induced ROS production, a redox-sensitive dye, CellROX^®^ Green (Thermo Fisher Scientific Inc.), and a red mitochondrial superoxide indicator, MitoSOX^®^ Red (Thermo Fisher Scientific Inc.), was used in accordance with the manufacturer’s instructions. The N2a cells were prepared in uncoated glass-bottomed microwells. Following plasmid transfection, CellROX^®^ Green and MitoSOX^®^ Red were added to the cell culture to a final concentration of 5 μM, and the cells were incubated for 30 min at 37°C. Fluorescent microscopy images were acquired using a confocal fluorescence microscope (LSM700; Carl Zeiss) and were analyzed using ZEN software (Carl Zeiss).

#### Quantitative RT-PCR

After 24 h of vector transfection into N2a cells, the cells were treated with SHED-CM (30%, 50%, 70%/24 h). These RNA sample were extracted and reverse transcription was performed using ReverTra Ace qPCR RT Master Mix in accordance with the manufacturer’s instructions (TOYOBO). qRT-PCR was performed using SYBR Green on a StepOne Real-Time PCR System according to the manufacturer’s instructions (Life Technologies). The sequences of gene-specific primer sets are shown in Table 1. The expression levels of mRNA were normalized using the expression levels of β-actin and Gapdh mRNA.

**TABLE 1:** Real time qPCR primers.

	Forward	Reverse
*Ho1*	5′-cac​gca​tat​acc​cgc​tac​ct-3′	5′-cca​gag​tgt​tca​ttc​gag​a-3′
*Nqo1*	5′-cgc​aga​cct​tgt​gat​att​cca​g-3′	5′-cgt​ttc​ttc​cat​cct​tcc​agg-3′
*Gclm*	5′-ttg​gag​ttg​cac​agc​tgg​att​c-3′	5′-tgg​ttt​tac​ctg​tgc​cca​ctg-3′
*Hspd1*	5′-gat​atg​gct​att​gct​act​ggt​ggt​gc-3′	5′-cct​aag​tca​tga​gct​tga​aca​tct​tc-3′
*Hspa1*	5′-tgg​tgc​agt​ccg​aca​tga​ag-3′	5′-gct​gag​aat​cgt​tga​agt​agg​c-3′
*Hsp90aa*	5′-atg​aca​gcg​gca​aag​aca​ag-3′	5′-agg​tcc​tcg​gag​tca​acc​ac-3′
*β-actin*	5′-cgt​tga​cat​ccg​taa​aga​cc-3′	5′-gct​agg​agc​cag​agc​agt​aa-3′
*Gapdh*	5′-cct​cgt​ccc​gta​gac​aaa​atg-3′	5′-tct​cca​ctt​tgc​cac​tgc​aa-3′

#### Phospho-Receptor Tyrosine Kinase Array

We conducted parallel determination of the relative level of tyrosine phosphorylation of human receptor tyrosine kinases (RTKs) (R&D Systems). The cells were treated with SHED-CM (70%) for 30 min. After treatment, the sample collection and storage conditions listed on the product sheet were used. The next day, the membrane was washed twice with wash buffer. The wash buffer was carefully removed and then diluted anti-phospho tyrosine-HRP was added. Finally, the cells were incubated in ECL prime (GE Healthcare) to generate HRP antibody chemiluminescence, which was detected by the aforementioned Fusion system.

#### Differentiation of Motor Neurons

We used iPSCs in which polycistronic vectors containing Lhx3, Ngn2, and Isl1 (LNI cassette) were provided by the Center for iPS Cell Research and Application. The cells were passaged using TrypLE (Thermo Fisher Scientific Inc.) every 7 days. When these cells were differentiated into motor neurons, they were plated onto Matrigel (Corning)-coated dishes with STEM Fit (Ajinomoto, Tokyo, Japan). The next day, all media were changed to Neurobasal Medium (Thermo Fisher Scientific Inc.), including an N2 supplement (Thermo Fisher Scientific) and doxycycline (1 mg/ml) (Takara), and the cells were cultured for 7 days. Generation and use of human iPSCs was approved by the Ethics Committees of the respective departments including Kyoto University. The study using iPS cells was approved by the Ethics Committees of Kyoto University (R0091, G259) and performed in accordance with the Ethical Guidelines for Medical and Health Research Involving Human Subjects in Japan as well as the Ethical Guidelines for Human Genome/Gene Analysis Research in Japan.

#### Experiment Using ALS Motor Neurons

iPSCs with the LNI cassette were dissociated to single cells using TrypLE (Thermo Fisher Scientific Inc.) and then plated onto Matrigel-coated 12-well plates (BD Biosciences) with Neurobasal Medium, including an N2 supplement and doxycycline (1 mg/ml), in which they were cultured for 7 days. When the cells had differentiated into motor neurons, SHED-CM was added and the cells were stained on day 14. SHED-CM and fibroblast-CM (Fb-CM) were concentrated by about 20-fold *via* ultrafiltration (for example, 4 ml was concentrated to 200 µl). In this iPS cell study, both concentrated CMs were treated with 25 µl in 1 ml of Neurobasal Medium including an N2 supplement and doxycycline (1 mg/ml). The number of surviving motor neurons was counted using staining for SMI-32.

#### Preparation of CM

SHED-CM and Fb-CM were provided by Tokushima University. Briefly, SHED and fibroblasts at 70%–80% confluence were washed with PBS, and the culture medium was replaced with serum-free DMEM. After 48-h incubation, the medium was collected and centrifuged for 4–5 min at 440 g. The supernatant was then collected and centrifuged for 1 min at 4°C and 17,400 g. All experiments on SHED-CM were approved by the Ethics Committees of Gifu Pharmaceutical University, Tokushima University (3,269-1) and performed in accordance with the Ethical Guidelines for Medical and Health Research Involving Human Subjects in Japan as well as the Ethical Guidelines for Human Genome/Gene Analysis Research in Japan.

#### Statistical Analysis

The results are presented as means ± standard error of the mean (SEM). Statistical comparisons were performed using either Student’s t-test or ANOVA followed by the Bonferroni/Dunn test for post-hoc comparisons (StatView, Abacus; Baltimore, MD, USA). *p* values <0.05 were considered statistically significant.

### Results

#### SHED-CM Suppresses Mutant SOD1 Intracellular Aggregates and Protects Against Mutant SOD1-Induced Neurotoxicity

**FIGURE 1:**
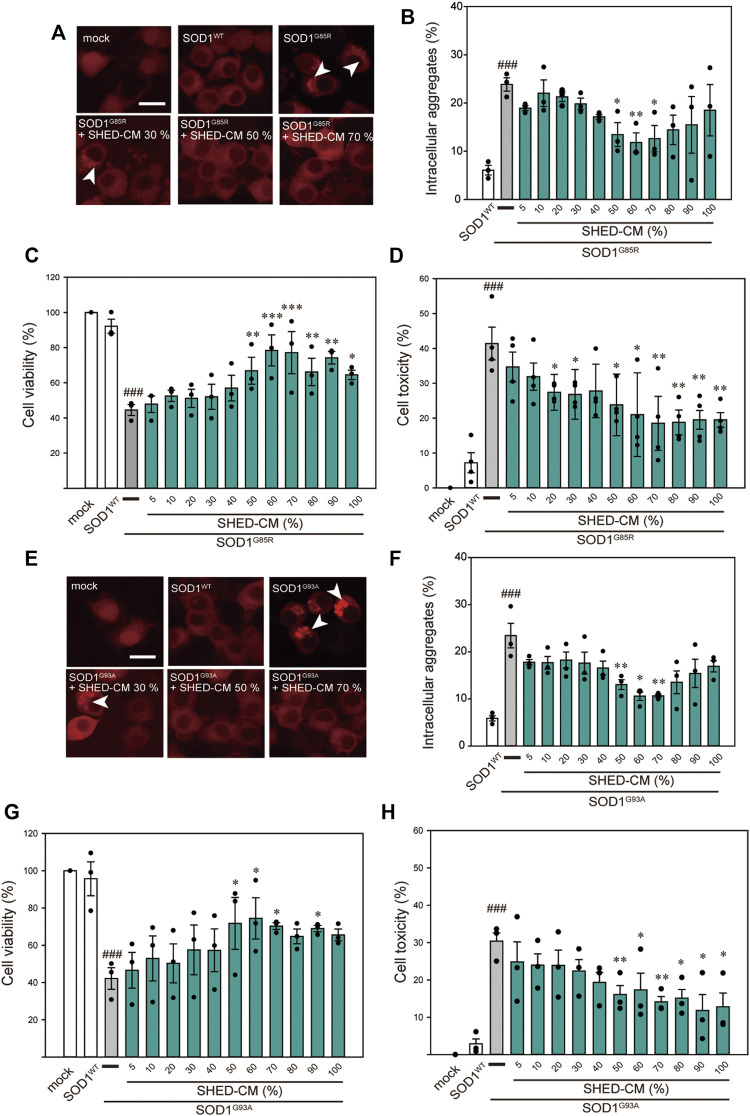
SHED-CM protects against SOD1^G85R^- and SOD1^G93A^-induced aggregation and neurotoxicity. **(A–E)** shows data on the G85R variant, whereas **(E–H)** shows data on the G93A variant. **(A and E):** Representative fluorescent microscopy images of N2a cells expressing mCherry-SOD1^G85R^ and mCherry-SOD1^G93A^ incubated for 24 h with SHED-CM (5%–100%). Arrow heads show SOD1 aggregates. **(B and F)**: Quantified data on intracellular SOD1 aggregates (expressed as means ± SEM of three independent experiments). In each experiment, at least 2,000 cells were counted. **(C and G):** Cell viability was measured via a CCK-8 assay. **(D and H):** Cell toxicity was measured via an LDH assay. N2a cells were differentiated under culture medium (2-mM dbcAMP and 2% FBS) in the presence or absence of SHED-CM (5%–100%/24 h). Results are presented as means ± SEM of three independent experiments or as a percentage of “mock” (mock = 100%). ###*p* < 0.001 vs. WT; ***p < 0.001, **p < 0.01, and *p < 0.05 vs. G85R and G93A. Scale bar: 10 µm.

Among the 150 types of pathogenic SOD1 mutations reported, the SOD1^G85R^ mutation is known to lead to rapid loss of motor neurons regardless of its enzymatic activity (Ripps, et al., 1995). Based on our previous study, SOD1^G85R^- and SOD1^G93A^-transfected N2a cells were created as mutant SOD1 models (Ueda T et al., 2019; Ueda T et al., 2018). These cells show accumulation of intracellular aggregates and decreased cell viability. Using these model cells, we examined the effect of SHED-CM against mutant SOD1-induced neurotoxicity. We confirmed that SHED-CM (50%–70%) decreased the accumulation of SOD1^G85R^ and SOD1^G93A^ aggregates, although the intensity of the fluorescence was not linear with respect to the quantity of proteins in aggregates (Figures 1A,B,E,F). To investigate the effect of SHED-CM against SOD1^G85R^- and SOD1^G93A^ -induced neurotoxicity, CCK-8 and LDH assays were performed (Figures 1C,D,G,H). We found that SHED-CM (50%–70%) prevented SOD1^G85R^- and SOD1^G93A^ -induced neurotoxicity; however, the protective effect of SHED-CM slightly decreased at higher concentrations. This may have been due to a lack of the nutrients that are required for survival *in vitro*. We also examined the effect of the serum-free culture supernatant of human skin fibroblasts (i.e., Fb-CM); we found that Fb-CM (30%) suppressed the intracellular aggregates of mutant SOD1, although not to the same extent as the suppression produced by SHED-CM. Furthermore, Fb-CM had no neuroprotective effect on cell viability (Supplementary Figure S1).

#### SHED-CM Suppresses Mutant SOD1-Related Oxidative Stress

**FIGURE 2:**
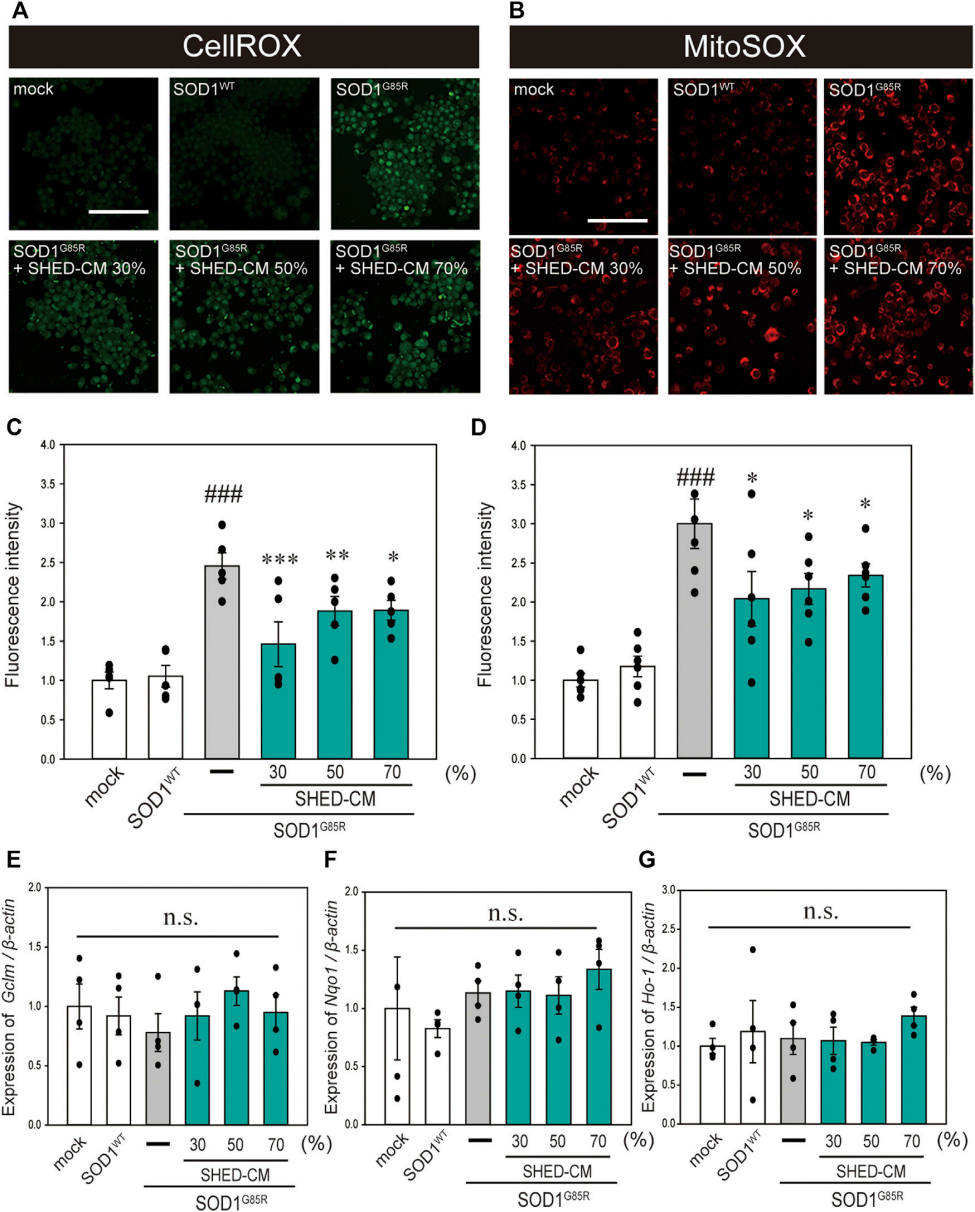
SHED-CM suppresses SOD1^G85R^-induced oxidative stress. **(A)**: N2a cells expressing mCherry-SOD1^G85R^ were treated with SHED-CM (30%, 50%, and 70%) for 24 h. Subsequently, CellROX Green was added to the cell culture to a final concentration of 5 μM and incubated for 30 min at 37°C. **(B)**: MitoSOX Red was added to the cell culture to a final concentration of 5 μM and incubated for 30 min at 37°C. **(C and D):** The relative fluorescence intensities of CellROX and MitoSOX were respectively quantified by computerized image analysis with Image J. Results are presented as the means ± SEM of three independent experiments, based on the fluorescence intensity of the “mock” (mock = 1). ###*p* < 0.001 vs. WT; ***p < 0.001, **p < 0.01, and *p < 0.05 vs. G85R. Scale bar: 100 µm. **(E–G)**: Expression of *Gclm, Nqo1, and Ho-1* are presented as a ratio of *β-actin*, respectively. N2a cells expressing mCherry-SOD1^G85R^ were treated with SHED-CM (30%, 50%, and 70%) for 24 h, and mRNA expressions were analyzed using a SYBR Green-based RT-qPCR assay. The expression levels of mRNAs were normalized to the expression level of β-actin mRNA. Results are presented as means ± SEM from three independent experiments based on the fluorescence intensity of the “mock” (mock = 1). n.s.: not significant.

In our previous study, oxidative stress was recorded at high levels in N2a cells with SOD1 pathogenic variants (Ueda, et al., 2018; Ueda, et al., 2019). Oxidative stress is a feature of ALS pathogenesis (Liu, et al., 2015; Bond, et al., 2018), and is related to increased production of ROS and oxidative damage to proteins (D’Amico, et al., 2013). In addition, suppression of oxidative stress is considered to inhibit the progression of ALS (Carrera-Juliá, et al., 2020). Therefore, we examined the effect of SHED-CM against mutant SOD1-related oxidative stress using CellROX Green MitoSOX Red. Results showed that SHED-CM treatment suppressed mutant SOD1-related oxidative stress (Figures 2A–D). To determine whether the suppression of oxidative stress was related to gene expression, real-time qPCR was performed for *GCLM, NQO1, and HO-1*, which are known as markers of antioxidative stress. Results showed that SHED-CM had no effect on the expression of these genes (Figures 2E–G).

#### SHED-CM Suppresses Mutant SOD1-Induced ER Stress and Increases HSP70 Levels

**FIGURE 3:**
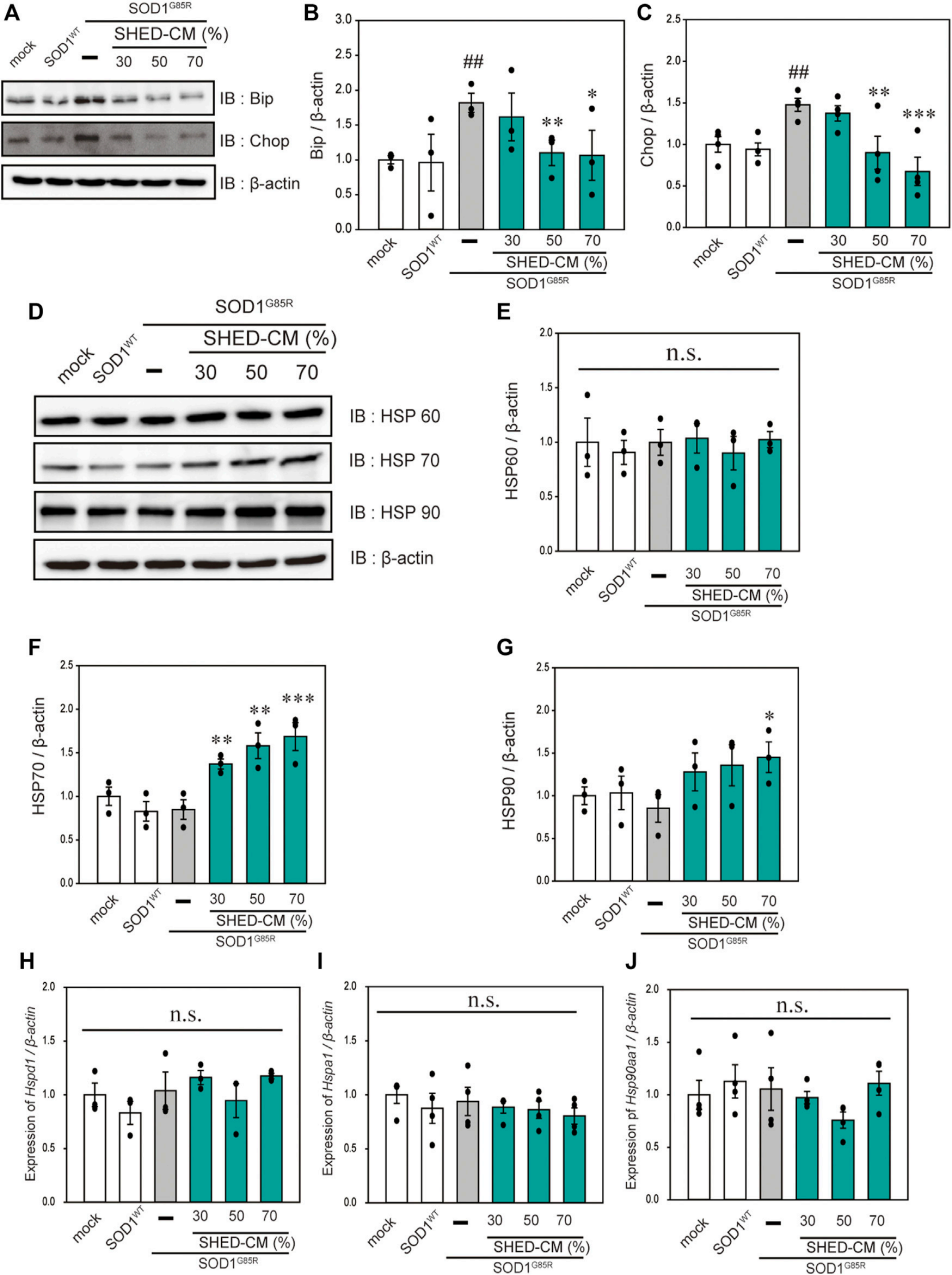
SHED-CM suppresses SOD1^G85R^ -induced ER stress and increases HSP70 levels. **(A)**: N2a cells expressing mCherry-SOD1G85R were treated with SHED-CM (30%, 50%, and 70%) for 24 h. Subsequently, immunoblot analysis of Bip and Chop was conducted in relation to ER stress. **(B and C)**: Densitometric quantification of Bip and Chop. ###*p* < 0.001 vs. WT; ***p < 0.001, **p < 0.01, and *p < 0.05 vs. G85R. **(D)**: Immunoblot analysis of HSP60, HSP70, and HSP90. **(E–G)**: Densitometric quantification of HSP60, HSP70, and HSP90. Results are presented as means ± SEM of three independent experiments based on the fluorescence intensity of the “mock” (mock = 1). ***p < 0.001, **p < 0.01, and *p < 0.05 vs. G85R. n.s.: not significant. **(H–J)**: N2a cells expressing mCherry-SOD1^G85R^ were treated with SHED-CM (30%, 50%, and 70%) for 24 h, HSP-related gene mRNA expression of HSP60, HSP70, and HSP90 (*Hspd1, Hspa1, and Hsp90aa1*, respectively) were analyzed using the SYBR Green-based RT-qPCR assay. The expression levels of mRNAs were normalized to the expression level of β-actin mRNA. Results are presented as means ± SEM of three independent experiments based on the fluorescence intensity of the “mock” (mock = 1). n.s.: not significant.

**FIGURE 4:**
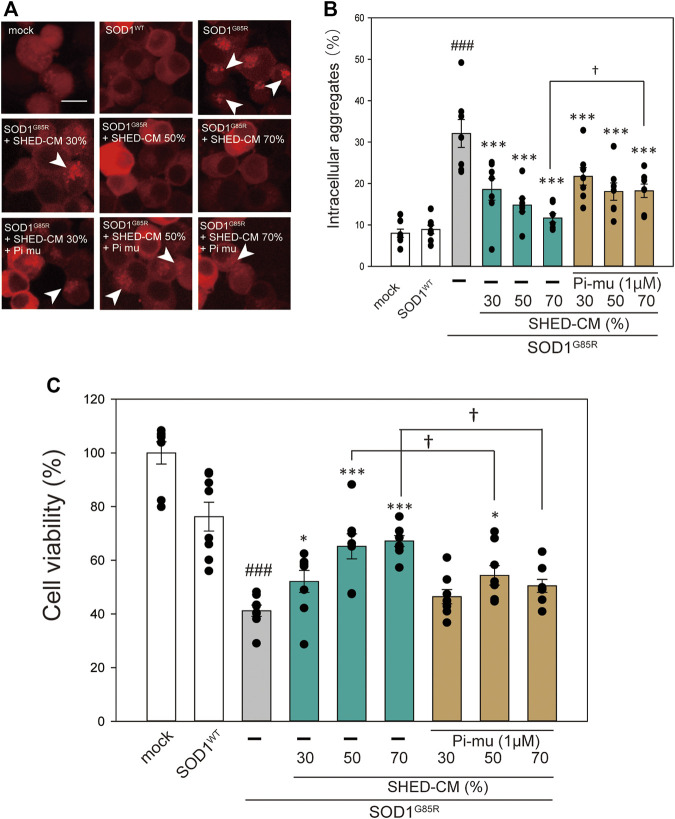
HSP70-related protective effects of SHED-CM. **(A)**: Representative fluorescent microscopy images of N2a cells expressing mCherry-SOD1^G85R^ incubated for 24 h with SHED-CM (30%, 50%, and 70%) and the HSP70 inhibitor Pifithrin mu (Pi mu) (1 μM/24 h). Arrow heads show SOD1 aggregates. **(B)**: Quantified data of intracellular SOD1 aggregates are expressed as means ± SEM of three independent experiments. In each experiment, at least 2,000 cells were counted. ###*p* < 0.001 vs. WT; ***p < 0.001, **p < 0.01, and *p < 0.05 vs. G85R; †*p* < 0.05 vs. G85R treatment of SHED-CM. Scale bar: 10 µm. **(C)**: mCherry-, mCherry-SOD1^WT^-, and mCherry-SOD1^G85R^-expressing N2a cells were differentiated in culture medium (2-mM dbcAMP and 2% FBS) in the presence or absence of SHED-CM (30%, 50%, and 70%/48 h) and the HSP 70 inhibitor (Pi mu) (1 μM/48 h). Cell viability was measured via a CCK-8 assay. Results are presented as means ± SEM of three independent experiments or as a percentage of the “mock” (mock = 100%). ###*p* < 0.001 vs. WT; ***p < 0.001, **p < 0.01, and *p < 0.05 vs. G85R; †*p* < 0.05.

In our previous study, we reported that ER stress is high in mutant SOD1 model cells (Ueda, et al., 2019). Additionally, ER stress is a known feature of ALS pathogenesis (Kikuchi, et al., 2006). Therefore, we examined the effect of SHED-CM against mutant SOD1-related ER stress. SHED-CM decreased mutant SOD1-induced ER stress (Figures 3A–C) and suppressed mutant SOD1 aggregates (Figure 1). To understand how SHED-CM suppresses these aggregates, we focused on HSPs, which are universally present in all cells and have a variety of functions. In particular, HSP expression levels are known to increase when cells are exposed to stresses such as heat and oxygen deprivation (Jacob, et al., 2017). Furthermore, HSPs such as HSP90, HSP70, HSP60, HSP40, and HSP27 play roles in maintaining the correct structure of intracellular proteins as chaperones. Several reports have shown the relationship between HSPs and misfolding proteins including SOD1, synuclein, and tau (Auluck, et al., 2002; Dedmon, et al., 2005; Sharp, et al., 2008). Here, we hypothesized that the effects of SHED-CM against mutant SOD1 involve HSPs; thus, we performed western blots for representative HSPs, i.e., HSP60, HSP70, and HSP90. Results showed that the expression of HSP70 in particular was increased by SHED-CM treatment (Figures 3D–G). To determine whether the increase in HSP70 was related to gene expression, real-time qPCR for *Hspd1, Hspa1, and Hsp90aa1* was performed. Data showed that SHED-CM had no effect on the expression of the HSP-related genes in the studies using β-actin mRNA (Figures 3H–J) and also using Gapdh mRNA (Supplementary Figure S2). To clarify the involvement of HSP70 in the protective effect of SHED-CM against mutant SOD1, intracellular aggregates and cell viability were examined using a HSP70 inhibitor (Pifithrin mu). Cotreatment with SHED-CM and the HSP70 inhibitor increased mutant SOD1-induced intracellular aggregates and attenuated the protective effect of SHED-CM (Figures 4A–C). These results indicate that the molecular chaperone HSP70 is partly responsible for the protective effect of SHED-CM.

#### SHED-CM has Neuroprotective Effects *via* IGF-1 Receptor

**FIGURE 5:**
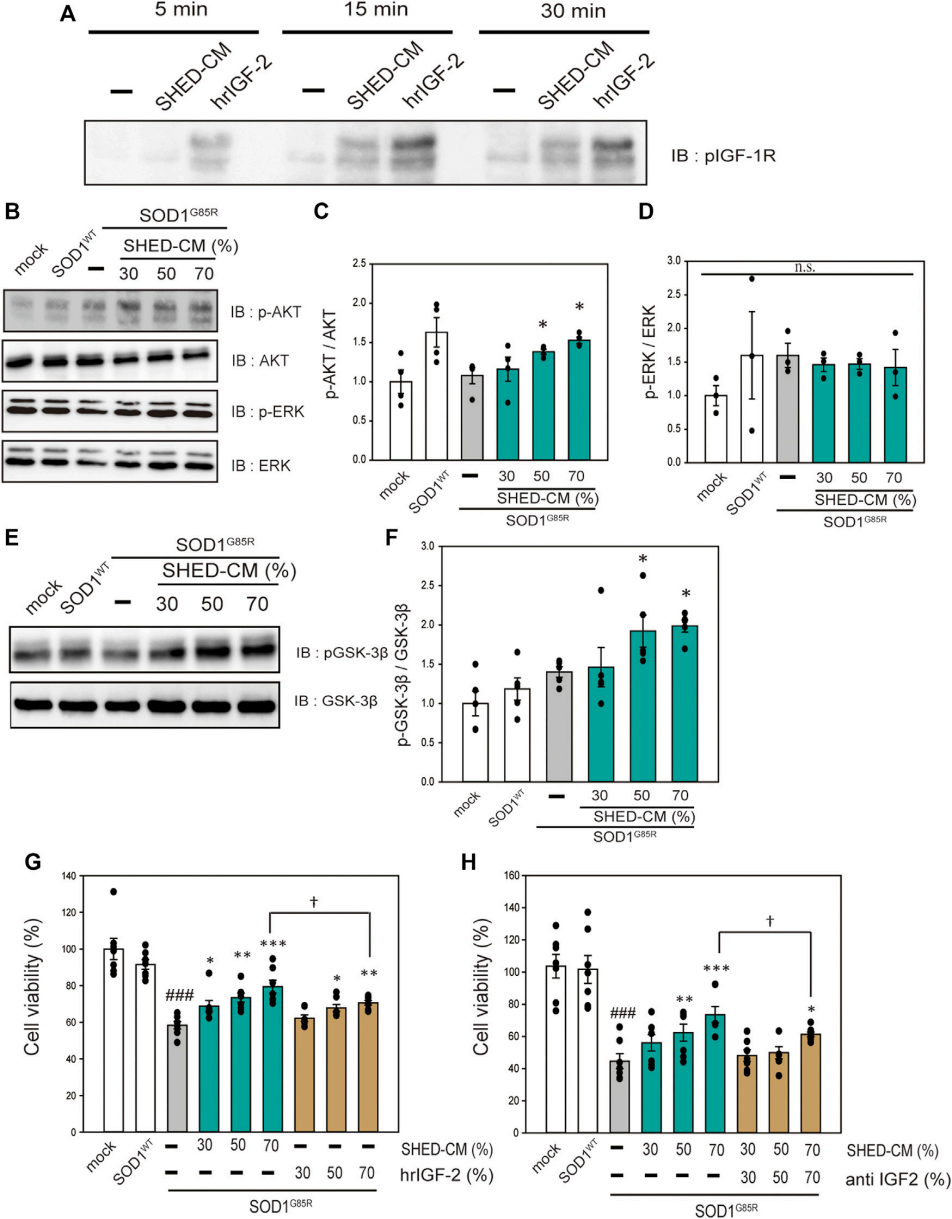
SHED-CM effects the activation of IGF-1 receptor. **(A)**: N2a cells were treated with SHED-CM (50%) for 5, 15, and 30 min. Subsequently, immunoblot analysis of p-IGF-1R was conducted. **(B and E)**: The lysates were analyzed by immunoblotting with antibodies for phosphorylated AKT (p-AKT), phosphorylated ERK (p-ERK), phosphorylated GSK-3β (p-GSK-3β), and β-actin. **(C, D, and F):** Densitometric quantification of p-AKT, p-ERK, and p-GSK-3β. Results are presented as means ± SEM of three independent experiments based on the fluorescence intensity of the “mock” (mock = 1). ***p < 0.001, **p < 0.01, and *p < 0.05 vs. G85R. **(G)**: N2a cells expressing mCherry-SOD1^G85R^ were treated with SHED-CM (30%, 50%, and 70%) and h-recombinant IGF-2 (same amount as included in SHED-CM) for 24 h. Cell viability was measured using a CCK-8 assay. **(H)**: N2a cells expressing mCherry-SOD1^G85R^ were treated with SHED-CM (30%, 50%, and 70%) and neutralizing anti-IGF-2 antibody for 24 h. Cell viability was measured *via* a CCK-8 assay. Results are presented as means ± SEM of three independent experiments based on the fluorescence intensity of the “mock” (mock = 100). ###*p* < 0.001 vs. WT; ***p < 0.001, **p < 0.01, and *p < 0.05 vs. G85R; †*p* < 0.05.

SHED-CM includes a variety of growth factors (Yamagata M et al., 2013). Therefore, to investigate the neuroprotective effects of SHED-CM in relation to receptor activation, an RTK array was performed to detect receptor phosphorylation. Among a number of receptors, IGF-1R tended to be activated; thus, western blotting was used to confirm the activation of IGF-1R by treatment with SHED-CM (Supplementary Figure S3 and Figure 5A). To identify the signal transduction pathway, we focused on AKT and ERK, which are downstream signal proteins of IGF-1R and are associated with ALS (Halon-Golabek, et al., 2018; Yin, et al., 2015; Ionescu, et al., 2019; Wang, et al., 2018). SHED-CM activated AKT and phosphorylated its downstream signal, GSK-3β (Figures 5B–F). AKT inactivates GSK-3β; previous studies have shown that phosphorylation of the AKT–GSK-3β pathway has neuroprotective effects in SOD1 mouse models (Allodi, et al., 2016). Therefore, the neuroprotective effect of SHED-CM is partly related to the phosphorylation of IGF-1R, AKT, and GSK-3β. Furthermore, to clarify the relationship between SHED-CM and IGF-1R, the effects of SHED-CM were also examined using human recombinant IGF2 (hrIGF2) and neutralizing IGF-2 antibodies. First, hrIGF2 showed a significant protective effect, although not as strong as the protective effect of SHED-CM (Figure 5G). Second, the protective effect of SHED-CM was suppressed by treatment with neutralizing IGF antibody (Figure 5H). These results suggest that IGF-1R may be involved in the protective effect of SHED-CM.

#### SHED-CM has a Protective Effect on iPS Cell-Derived Motor Neurons

**FIGURE 6:**
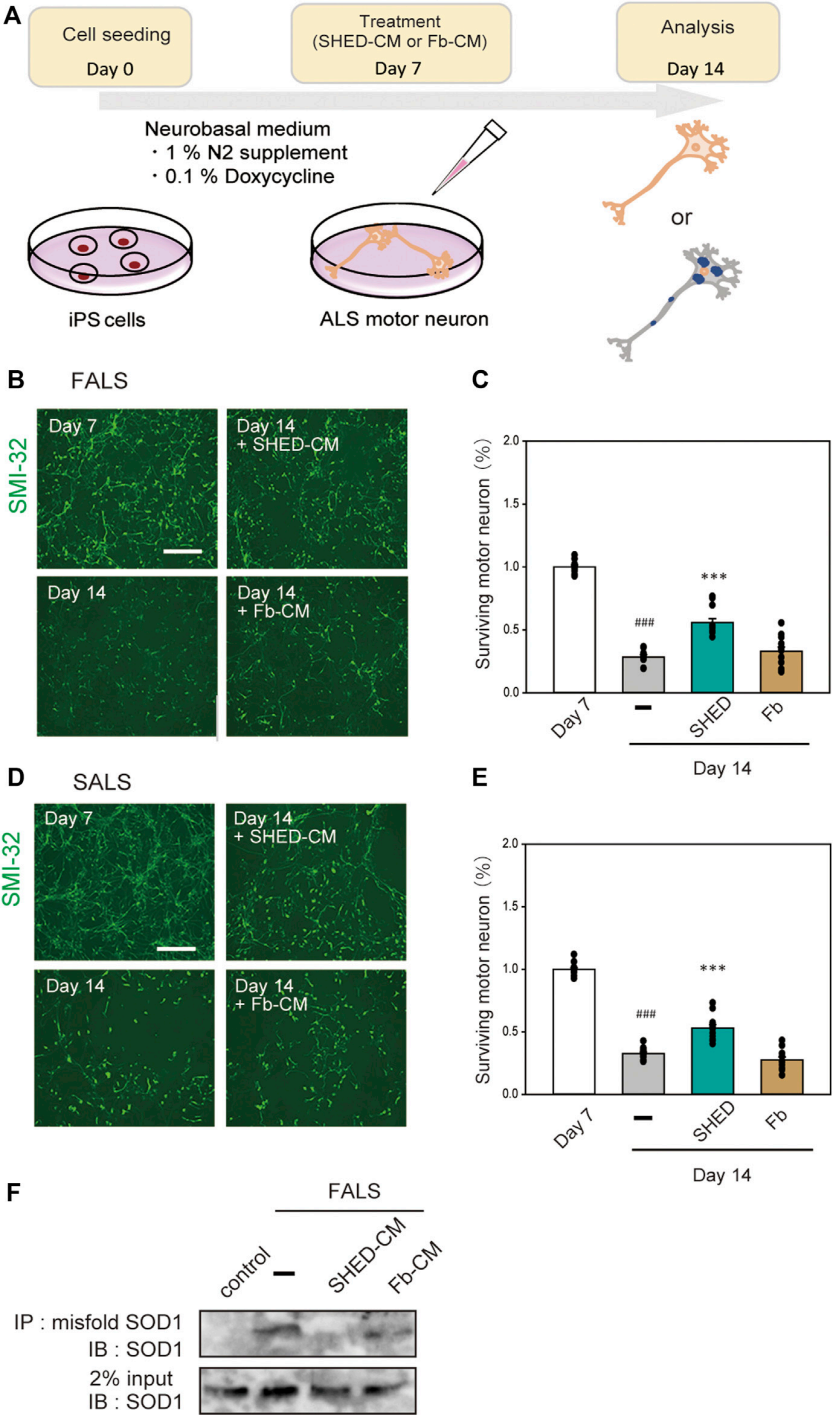
Neuroprotection of ALS motor neurons using (or treated with) SHED-CM. **(A)**: The scheme of this study. **(B and D)**: Generation of motor neurons from iPSCs derived from ALS patient. Representative images of ALS motor neurons on day 7 and day 14 after treatment with vehicle, ultrafiltered SHED-CM (50%), or Fb-CM (50%). Treatment with SHED-CM increased survival of ALS motor neurons compared with the survival of vehicle-treated ALS motor neurons. Scale bar: 200 µm. **(C and E)**: Cell viability was measured by staining SMI-32. Results are presented as means ± SEM of three independent experiments based on the fluorescence intensity of the “mock” (mock = 100). ###*p* < 0.001 vs. Day 7; ***p < 0.001 vs. Day 14. Scale bar: 200 µm. **(F)**: Accumulation of misfolded SOD1 protein in ALS patient iPSC-derived motor neurons was detected using an immunoprecipitation assay.

To examine the effect of SHED-CM on human-derived cells, we used iPS cells derived from FALS (F144LVX) patients and differentiated them into motor neurons over 7 days (Imamura, et al., 2017). These cells expressed high levels of SMI-32, a marker for motor neurons. After 7 days of differentiation, SHED-CM and Fb-CM were added to the culture medium for another 7 days; at day 14, the surviving motor neurons were measured using immunostaining for SMI-32 (Figure 6A). However, since the culture mediums of iPS cells and SHED are different, SHED-CM was concentrated by ∼20-fold by ultrafiltration. Results showed that most of the cells in the untreated group were dead by day 14, whereas the survival rate in the SHED-CM-treated group recovered to ∼60% (Figures 6B,C). In addition, iPS cells derived from a sporadic ALS (SALS) patient were also used, and we found SHED-CM treatment had a similar efficiency against SALS as was observed against FALS (Figures 6D,E). To further examine the protective effect of SHED, the accumulation of abnormal proteins in motor neurons was detected by immunoprecipitation using an antibody that specifically recognizes misfolding SOD1. Results showed that SHED-CM reduced misfolding SOD1 in FALS (Figure 6F). These results suggest that SHED-CM is effective not only against FALS but also against SALS; therefore, it has the potential to be an effective therapeutic medicine.

## Discussion

Here, we reveal that SHED-CM is a promising candidate for the treatment of ALS using iPS cells. Previously, we created a mutant SOD1-induced neurotoxicity model using N2a cells that resembles the characteristics of motor neurons of patients. In our prior studies, we identified new candidate processes such as activation of autophagy *via* the AMPK-mTOR pathway and antioxidant effects that can remove various ROS (Ueda, et al., 2017; Ueda, et al., 2018). The approved drugs currently used to treat ALS in Japan, e.g., edaravone and riluzole, have extremely limited effects (Lacomblez, et al., 1996). To solve this problem, we have attempted to identify drugs that produce additive effects. Here, we focused on SHED, which release factors such as neurotrophic factors (Matsubara, et al., 2015) and biometals (unpublished data). The efficacy of SHED and SHED-CM are now being actively studied worldwide (Shimojima, et al., 2016; Shi, et al., 2020). However, the effects of SHED-CM on the nervous system, especially in ALS, had previously not been investigated. In the present study, SHED-CM exhibited protective effects in mutant SOD1-transfected N2a cells and ALS patient-derived iPS cells.

Mutant SOD1 accounts for about 20% of FALS in Japan and intracellular aggregates are observed in the motor neurons of these patients (Grad, et al., 2014). Inhibition of such aggregates is one strategy for ALS treatment (Watanabe, et al., 2014). In the current study, SHED-CM was shown to reduce mutant SOD1 intracellular aggregates in both the N2a cell model and iPS cells. HSPs are involved in inhibiting this aggregation (Auluck, et al., 2002; Dedmon, et al., 2005; Sharp, et al., 2008). Previous reports showed that arimoclomol, a coinducer of HSP responses, recovered motor function and survival in SOD1Tg mice (Kieran, et al., 2004; Kalmar, et al., 2008). In addition, a double-blind placebo study using arimoclomol in ALS patients with SOD1 mutants showed that the therapeutic effects were not detected at a statistically significant level, although the treatment was potentially effective for ALS patients with several mutations (Benatar, et al., 2018). In our present study, HSP70 levels were increased by SHED-CM treatment, while HSP90 also showed an increasing trend. These results suggest that HSPs are partly involved in the protective effect of SHED-CM against mutant SOD1. However, the study using a HSP 70 inhibitor may not be fully sufficient data, HSP70 is thought to be partially involved in the protective effect of SHED-CM against mutant SOD1. I addition, previous studies showed that HSPs have protective effects not only against mutant SOD1 but also against TDP-43 aggregates, which are known to accumulate in the motor neurons of SALS patients (Chen, et al., 2016). Considering these results, SHED-CM seems to produce protective effects on the motor neurons SALS patients *via* the same mechanism by which such effects are produced in FALS patients.

Based on these results of Cellrox and Mitosox, we hypothesized that SHED-CM increased the expression of the antioxidant gene (*Gclm, Nqo1, and Ho1*). However, SHED-CM had no effect on the expression of antioxidant genes (Figures 2E–G). The reason for no changes in the antioxidant gene is thought to be due to that the inhibition of aggregate formation reduces oxidative stress. Several reports have shown the relationship between HSPs and misfolding proteins including SOD1, synuclein, and tau (Auluck, et al., 2002; Dedmon, et al., 2005; Sharp, et al., 2008). In addition, SHED-CM increased the protein level of HSP70 in this study. Therefore, we thought that SHED-CM suppresses oxidative stress *via* the HSP70 without the intervention of genes such as *Gclm, Nqo1, and Ho1.*

To investigate the downstream of signals induced by SHED-CM, we used an RTK array to determine which receptors were phosphorylated by SHED-CM. We found that SHED-CM activated various receptors (Supplementary Figure S3). Previous reports showed that activation of IGF-1R prolongs survival in a mouse ALS model (Allodi et al., 2016; Wen, et al., 2019). Indeed, IGF-1R is a key molecule; our results suggest that IGF-1R, AKT, and GSK3-β pathways contribute significantly to cell survival. Many other studies have reported the existence of a relationship between GSK3-β and mutant SOD1. The use of GSK-3β inhibitors prevents apoptosis and ultimately suppresses the onset of symptoms and disease progression in SOD1Tg mice (Koh, et al., 2007; Ahn, et al., 2012). These results suggest that the phosphorylation of GSK-3β is partly involved in the observed protective effects of SHED-CM. In this study, we reveal the activation of IGF receptor and HSP 70 was partly related to neuroprotective effects against the neurotoxicity of mutant SOD1. However, these protective effects are less effective than those of SHED-CM (Figure 4, Figures 5G,H).

Previous studies showed that HGF was shown to increase the number and diameter of motor neurons, restore the shape of the neuromuscular junction, improve motor function, and prolong survival rate in SOD1Tg mice (Lee, et al., 2019; Lee, et al., 2019). In the current study, SHED-CM did not activate the HGF receptor in N2a cells (Supplementary Figure S3). In addition, a previous report showed that stem cell-derived CM increases the expression of antioxidant factors (Ra, et al., 2020). However, SHED-CM could not increase in the expression of antioxidant factors such as *Gclm, Nqo1, and Ho-1* in our study. We suppose that the reactions and pathways induced by SHED-CM are different among kinds of cells, injuries and species, because SHED-CM contains various neurotrophic factors and biometals, and additionally unknown substances.

A previous study has reported that the effect of DPC-derived CM on mutant SOD1Tg mice (Wang, et al., 2019) Historically, however, many differences in the expression of receptors, reactions and toxicity exist between humans and mice. Recently, iPS cells have been used in drug discovery research for ALS. Consequently, ropinirole, a medicine used to treat Parkinson’s disease, was identified as a novel therapeutic drug for ALS (Okano, et al., 2020). Therefore, we used iPS cells to examine the protective effects of SHED-CM from a clinical perspective. This is the first study in which SHED-CM and iPS cells were used against mutant SOD1. The iPS cells used here were differentiated into motor neurons in 7 days and showed characteristic symptoms of FALS and SALS.

There are some limitations to our research. For control, in future, we think it is necessary to study the isogenic control genetically corrected usingCRISPR-Cas9 technique). Although we examined the efficacy of SHED-CM on some motor neurons *in vitro*, additional patient-derived iPS cells with other variants should be examined to produce definitive conclusions. Furthermore, in future research, the efficacy of SHED-CM should be evaluated not only in neurons but also in glial cells *in vivo*. *In vivo* studied might be also necessary for clinical trails. Taken together, our iPS study using SHED-CM is a pioneering and promising study for ALS. Currently, the two research in which iPS cells are used to identify new medicines for ALS have been clinically developed in Japan (Okano H et al., 2020, Imamura K et al., 2017).

Few clinical trials in which SHED was used have been reported to date. However, many studies have been conducted for the purpose of developing the clinical application of SHED. Now clinical application of SHED into patients is a focus of regenerative medicine (Taghipour, et al., 2012; Fujii, et al., 2015). Indeed, SHED exists in young human bodies. In addition, long-term monitoring of SHED treatment shows that it is safe and does not produce adverse reactions (Nakashima, et al., 2017; Nakashima, et al., 2017). Moreover, SHED can differentiate into various cells including chondrocytes, endothelial cells, and functionally and structurally active neurons (Miura, et al., 2003; Fujii, et al., 2015). Therefore, SHED has great potential for use in stem cell therapy. In the future, SHED and SHED-CM are expected to be developed as clinical grade agents that could be widely used against various diseases.

## Data Availability

The raw data supporting the conclusion of this article will be made available by the authors, without undue reservation.

## References

[B1] Ahn S. W. Kim J. E. Park K. S. Choi W. J. Hong Y. H. Kim S. M. (2012). The Neuroprotective Effect of the GSK-3β Inhibitor and Influence on the Extrinsic Apoptosis in the ALS Transgenic Mice. J. Neurol. Sci. 320 (1-2), 1–5. 10.1016/j.jns.2012.05.038 22698482

[B2] Allodi I. Comley L. Nichterwitz S. Nizzardo M. Simone C. Benitez J. A. (2016). Differential Neuronal Vulnerability Identifies IGF-2 as a Protective Factor in ALS. Sci. Rep. 6, 25960. 10.1038/srep25960 27180807 PMC4867585

[B3] Auluck P. K. Chan H. Y. Trojanowski J. Q. Lee V. M. Bonini N. M. (2002). Chaperone Suppression of Alpha-Synuclein Toxicity in a Drosophila Model for Parkinson's Disease. Science 295 (5556), 865–868. 10.1126/science.1067389 11823645

[B4] Benatar M. Wuu J. Andersen P. M. Atassi N. David W. Cudkowicz M. (2018). Randomized, Double-Blind, Placebo-Controlled Trial of Arimoclomol in Rapidly Progressive SOD1 ALS. Neurology 90 (7), e565–e574. 10.1212/WNL.0000000000004960 29367439 PMC5818014

[B5] Bond L. Bernhardt K. Madria P. Sorrentino K. Scelsi H. Mitchell C. S. (2018). A Metadata Analysis of Oxidative Stress Etiology in Preclinical Amyotrophic Lateral Sclerosis: Benefits of Antioxidant Therapy. Front. Neurosci. 12, 10. 10.3389/fnins.2018.00010 29416499 PMC5787557

[B6] Bruijn L. I. Becher M. W. Lee M. K. Anderson K. L. Jenkins N. A. Copeland N. G. (1997). ALS-linked SOD1 Mutant G85R Mediates Damage to Astrocytes and Promotes Rapidly Progressive Disease with SOD1-Containing Inclusions. Neuron 18 (2), 327–338. 10.1016/s0896-6273(00)80272-x 9052802

[B7] Carrera-Juliá S. Moreno M. L. Barrios C. de la Rubia Ortí J. E. Drehmer E. (2020). Antioxidant Alternatives in the Treatment of Amyotrophic Lateral Sclerosis: A Comprehensive Review. Front. Physiol. 11, 63. 10.3389/fphys.2020.00063 32116773 PMC7016185

[B8] Chen H. J. Mitchell J. C. Novoselov S. Miller J. Nishimura A. L. Scotter E. L. (2016). The Heat Shock Response Plays an Important Role in TDP-43 Clearance: Evidence for Dysfunction in Amyotrophic Lateral Sclerosis. Brain 139 (5), 1417–1432. 10.1093/brain/aww028 26936937 PMC4845254

[B9] Cheroni C. Marino M. Tortarolo M. Veglianese P. De Biasi S. Fontana E. (2009). Functional Alterations of the Ubiquitin-Proteasome System in Motor Neurons of a Mouse Model of Familial Amyotrophic Lateral Sclerosis. Hum. Mol. Genet. 18 (1), 82–96. 10.1093/hmg/ddn319 18826962 PMC3298865

[B10] D'Amico E. Factor-Litvak P. Santella R. M. Mitsumoto H. (2013). Clinical Perspective on Oxidative Stress in Sporadic Amyotrophic Lateral Sclerosis. Free Radic. Biol. Med. 65, 509–527. 10.1016/j.freeradbiomed.2013.06.029 23797033 PMC3859834

[B11] Dedmon M. M. Christodoulou J. Wilson M. R. Dobson C. M. (2005). Heat Shock Protein 70 Inhibits Alpha-Synuclein Fibril Formation via Preferential Binding to Prefibrillar Species. J. Biol. Chem. 280 (15), 14733–14740. 10.1074/jbc.M413024200 15671022

[B12] Fujii H. Matsubara K. Sakai K. Ito M. Ohno K. Ueda M. (2015). Dopaminergic Differentiation of Stem Cells from Human Deciduous Teeth and Their Therapeutic Benefits for Parkinsonian Rats. Brain Res. 1613, 59–72. 10.1016/j.brainres.2015.04.001 25863132

[B13] Grad L. I. Yerbury J. J. Turner B. J. Guest W. C. Pokrishevsky E. O'Neill M. A. (2014). Intercellular Propagated Misfolding of Wild-type Cu/Zn Superoxide Dismutase Occurs via Exosome-dependent and -independent Mechanisms. Proc. Natl. Acad. Sci. U S A. 111 (9), 3620–3625. 10.1073/pnas.1312245111 24550511 PMC3948312

[B14] Gurney M. E. Pu H. Chiu A. Y. Dal Canto M. C. Polchow C. Y. Alexander D. D. (1994). Motor Neuron Degeneration in Mice that Express a Human Cu,Zn Superoxide Dismutase Mutation. Science 17264 (5166), 1772–1775. 10.1126/science.8209258 8209258

[B15] Halon-Golabek M. Borkowska A. Kaczor J. J. Ziolkowski W. Flis D. J. Knap N. (2018). hmSOD1 Gene Mutation-Induced Disturbance in Iron Metabolism Is Mediated by Impairment of Akt Signalling Pathway. J. Cachexia Sarcopenia Muscle 9 (3), 557–569. 10.1002/jcsm.12283 29380557 PMC5989766

[B16] Imamura K. Izumi Y. Watanabe A. Tsukita K. Woltjen K. Yamamoto T. (2017). The Src/c-Abl Pathway Is a Potential Therapeutic Target in Amyotrophic Lateral Sclerosis. Sci. Transl. Med. 9 (391), eaaf3962. 10.1126/scitranslmed.aaf3962 28539470

[B17] Inoue T. Sugiyama M. Hattori H. Wakita H. Wakabayashi T. Ueda M. (2013). Stem Cells from Human Exfoliated Deciduous Tooth-Derived Conditioned Medium Enhance Recovery of Focal Cerebral Ischemia in Rats. Tissue Eng. Part. A. 19 (1-2), 24–29. 10.1089/ten.TEA.2011.0385 22839964 PMC3530935

[B18] Ionescu A. Gradus T. Altman T. Maimon R. Saraf Avraham N. Geva M. (2019). Targeting the Sigma-1 Receptor via Pridopidine Ameliorates Central Features of ALS Pathology in a SOD1G93A Model. Cell Death Dis 10 (3), 210. 10.1038/s41419-019-1451-2 30824685 PMC6397200

[B19] Izumoto-Akita T. Tsunekawa S. Yamamoto A. Uenishi E. Ishikawa K. Ogata H. (2015). Secreted Factors from Dental Pulp Stem Cells Improve Glucose Intolerance in Streptozotocin-Induced Diabetic Mice by Increasing Pancreatic β-cell Function. BMJ Open Diabetes Res. Care 3 (1), e000128. 10.1136/bmjdrc-2015-000128 PMC4611480 26504525

[B20] Jacob P. Hirt H. Bendahmane A. (2017). The Heat-Shock Protein/chaperone Network and Multiple Stress Resistance. Plant Biotechnol. J. 15 (4), 405–414. 10.1111/pbi.12659 27860233 PMC5362687

[B21] Julien J. P. (2001). Amyotrophic Lateral Sclerosis. Unfolding the Toxicity of the Misfolded. Cell 104 (4), 581–591. 10.1016/s0092-8674(01)00244-6 11239414

[B22] Kalmar B. Novoselov S. Gray A. Cheetham M. E. Margulis B. Greensmith L. (2008). Late Stage Treatment with Arimoclomol Delays Disease Progression and Prevents Protein Aggregation in the SOD1 Mouse Model of ALS. J. Neurochem. 107 (2), 339–350. 10.1111/j.1471-4159.2008.05595.x 18673445

[B23] Kieran D. Kalmar B. Dick J. R. Riddoch-Contreras J. Burnstock G. Greensmith L. (2004). Treatment with Arimoclomol, a Coinducer of Heat Shock Proteins, Delays Disease Progression in ALS Mice. Nat. Med. 10 (4), 402–405. 10.1038/nm1021 15034571

[B24] Kikuchi H. Almer G. Yamashita S. Guégan C. Nagai M. Xu Z. (2006). Spinal Cord Endoplasmic Reticulum Stress Associated with a Microsomal Accumulation of Mutant Superoxide Dismutase-1 in an ALS Model. Proc. Natl. Acad. Sci. U S A. 103 (15), 6025–6030. 10.1073/pnas.0509227103 16595634 PMC1458691

[B25] Koh S. H. Kim Y. Kim H. Y. Hwang S. Lee C. H. Kim S. H. (2007). Inhibition of Glycogen Synthase Kinase-3 Suppresses the Onset of Symptoms and Disease Progression of G93A-SOD1 Mouse Model of ALS. Exp. Neurol. 205 (2), 336–346. 10.1016/j.expneurol.2007.03.004 17433298

[B26] Kondo T. Imamura K. Funayama M. Tsukita K. Miyake M. Ohta A. (2017). iPSC-Based Compound Screening and In Vitro Trials Identify a Synergistic Anti-amyloid β Combination for Alzheimer's Disease. Cell Rep 21 (8), 2304–2312. 10.1016/j.celrep.2017.10.109 29166618

[B27] Lacomblez L. Bensimon G. Leigh P. N. Guillet P. Meininger V. (1996). Dose-ranging Study of Riluzole in Amyotrophic Lateral Sclerosis. Amyotrophic Lateral Sclerosis/riluzole Study Group II. Lancet 347 (9013), 1425–1431. 10.1016/s0140-6736(96)91680-3 8676624

[B28] Lee S. H. Kim S. Lee N. Lee J. Yu S. S. Kim J. H. (2019b). Intrathecal Delivery of Recombinant AAV1 Encoding Hepatocyte Growth Factor Improves Motor Functions and Protects Neuromuscular System in the Nerve Crush and SOD1-G93a Transgenic Mouse Models. Acta Neuropathol. Commun. 7 (1), 96. 10.1016/j.bbrc.2019.07.10510.1186/s40478-019-0737-z 31189468 PMC6563368

[B29] Lee S. H. Lee N. Kim S. Lee J. Choi W. Yu S. S. (2019a). Intramuscular Delivery of HGF-Expressing Recombinant AAV Improves Muscle Integrity and Alleviates Neurological Symptoms in the Nerve Crush and SOD1-G93a Transgenic Mouse Models. Biochem. Biophys. Res. Commun. 517 (3), 452–457. 10.1016/j.bbrc.2019.07.105 31376938

[B30] Ling S. C. Polymenidou M. Cleveland D. W. (2013). Converging Mechanisms in ALS and FTD: Disrupted RNA and Protein Homeostasis. Neuron 79 (3), 416–438. 10.1016/j.neuron.2013.07.033 23931993 PMC4411085

[B31] Liu K. X. Edwards B. Lee S. Finelli M. J. Davies B. Davies K. E. (2015). Neuron-specific Antioxidant OXR1 Extends Survival of a Mouse Model of Amyotrophic Lateral Sclerosis. Brain 138 (5), 1167–1181. 10.1093/brain/awv039 25753484 PMC4407188

[B32] Mathis S. Goizet C. Soulages A. Vallat J. M. Masson G. L. (2019). Genetics of Amyotrophic Lateral Sclerosis: A Review. J. Neurol. Sci. 399, 217–226. 10.1016/j.jns.2019.02.030 30870681

[B33] Matsubara K. Matsushita Y. Sakai K. Kano F. Kondo M. Noda M. (2015). Secreted Ectodomain of Sialic Acid-Binding Ig-like Lectin-9 and Monocyte Chemoattractant Protein-1 Promote Recovery after Rat Spinal Cord Injury by Altering Macrophage Polarity. J. Neurosci. 35 (6), 2452–2464. 10.1523/JNEUROSCI.4088-14.2015 25673840 PMC6605605

[B34] Mita T. Furukawa-Hibi Y. Takeuchi H. Hattori H. Yamada K. Hibi H. (2015). Conditioned Medium from the Stem Cells of Human Dental Pulp Improves Cognitive Function in a Mouse Model of Alzheimer's Disease. Behav. Brain Res. 293, 189–197. 10.1016/j.bbr.2015.07.043 26210934

[B35] Miura M. Gronthos S. Zhao M. Lu B. Fisher L. W. Robey P. G. (2003). SHED: Stem Cells from Human Exfoliated Deciduous Teeth. Proc. Natl. Acad. Sci. U S A. 100 (10), 5807–5812. 10.1073/pnas.0937635100 12716973 PMC156282

[B36] Nakashima M. Iohara K. Murakami M. Nakamura H. Sato Y. Ariji Y. (2017). Pulp Regeneration by Transplantation of Dental Pulp Stem Cells in Pulpitis: A Pilot Clinical Study. Stem Cel Res. Ther. 8 (1), 61. 10.1186/s13287-017-0506-5 PMC5345141 28279187

[B37] Nakashima M. Iohara K. (2017). Recent Progress in Translation from Bench to a Pilot Clinical Study on Total Pulp Regeneration. J. Endod. 43 (9S), S82–S86. 10.1016/j.joen.2017.06.014 28778509

[B38] Norris S. P. Likanje M. N. Andrews J. A. (2020). Amyotrophic Lateral Sclerosis: Update on Clinical Management. Curr. Opin. Neurol. 33 (5), 641–648. 10.1097/WCO.0000000000000864 32868602

[B39] Okano H. Yasuda D. Fujimori K. Morimoto S. Takahashi S. (2020). Ropinirole, a New ALS Drug Candidate Developed Using iPSCs. Trends Pharmacol. Sci. 41 (2), 99–109. 10.1016/j.tips.2019.12.002 31926602

[B40] Ra K. Oh H. J. Kim G. A. Kang S. K. Ra J. C. Lee B. C. (2020). High Frequency of Intravenous Injection of Human Adipose Stem Cell Conditioned Medium Improved Embryo Development of Mice in Advanced Maternal Age through Antioxidant Effects. Animals (Basel) 10 (6), 978. 4. 10.3390/ani10060978 PMC7341498 32512813

[B41] Ripps M. E. Huntley G. W. Hof P. R. Morrison J. H. Gordon J. W. (1995). Transgenic Mice Expressing an Altered Murine Superoxide Dismutase Gene Provide an Animal Model of Amyotrophic Lateral Sclerosis. Proc. Natl. Acad. Sci. U S A. 92 (3), 689–693. 10.1073/pnas.92.3.689 7846037 PMC42685

[B42] Sharp P. S. Akbar M. T. Bouri S. Senda A. Joshi K. Chen H. J. (2008). Protective Effects of Heat Shock Protein 27 in a Model of ALS Occur in the Early Stages of Disease Progression. Neurobiol. Dis. 30 (1), 42–55. 10.1016/j.nbd.2007.12.002 18255302

[B43] Shi P. Gal J. Kwinter D. M. Liu X. Zhu H. (2010). Mitochondrial Dysfunction in Amyotrophic Lateral Sclerosis. Biochim. Biophys. Acta 1802 (1), 45–51. 10.1016/j.bbadis.2009.08.012 19715760 PMC2790551

[B44] Shi X. Mao J. Liu Y. (2020). Pulp Stem Cells Derived from Human Permanent and Deciduous Teeth: Biological Characteristics and Therapeutic Applications. Stem Cell Transl. Med. 9 (4), 445–464. 10.1002/sctm.19-0398 PMC7103623 31943813

[B45] Shimojima C. Takeuchi H. Jin S. Parajuli B. Hattori H. Suzumura A. (2016). Conditioned Medium from the Stem Cells of Human Exfoliated Deciduous Teeth Ameliorates Experimental Autoimmune Encephalomyelitis. J. Immunol. 196 (10), 4164–4171. 10.4049/jimmunol.1501457 27053763

[B46] Taghipour Z. Karbalaie K. Kiani A. Niapour A. Bahramian H. Nasr-Esfahani M. H. (2012). Transplantation of Undifferentiated and Induced Human Exfoliated Deciduous Teeth-Derived Stem Cells Promote Functional Recovery of Rat Spinal Cord Contusion Injury Model. Stem Cell Dev 21 (10), 1794–1802. 10.1089/scd.2011.0408 21970342

[B47] Ueda T. Inden M. Asaka Y. Masaki Y. Kurita H. Tanaka W. (2018). Effects of Gem-Dihydroperoxides against Mutant Copper-zinc S-uperoxide D-ismutase-M-ediated N-eurotoxicity. Mol. Cel. Neurosci. 92, 177–184. 10.1016/j.mcn.2018.09.001 30193933

[B48] Ueda T. Inden M. Shirai K. Sekine S. I. Masaki Y. Kurita H. (2017). The Effects of Brazilian green Propolis that Contains Flavonols against Mutant Copper-Zinc Superoxide Dismutase-Mediated Toxicity. Sci. Rep. 7 (1), 2882. 10.1038/s41598-017-03115-y 28588226 PMC5460160

[B49] Ueda T. Ito T. Kurita H. Inden M. Hozumi I. (2019). p-Coumaric Acid Has Protective Effects against Mutant Copper-Zinc Superoxide Dismutase 1 via the Activation of Autophagy in N2a Cells. Int. J. Mol. Sci. 20 (12), 2942. 10.3390/ijms20122942 PMC6628046 31208129

[B51] Wakayama H. Hashimoto N. Matsushita Y. Matsubara K. Yamamoto N. Hasegawa Y. (2015). Factors Secreted from Dental Pulp Stem Cells Show Multifaceted Benefits for Treating Acute Lung Injury in Mice. Cytotherapy 17 (8), 1119–1129. 10.1016/j.jcyt.2015.04.009 26031744

[B52] Wang J. Zuzzio K. Walker C. L. (2019). Systemic Dental Pulp Stem Cell Secretome Therapy in a Mouse Model of Amyotrophic Lateral Sclerosis. Brain Sci. 9 (7), 165. 10.3390/brainsci9070165 PMC6680809 31337114

[B53] Wang T. H. Wang S. Y. Wang X. D. Jiang H. Q. Yang Y. Q. Wang Y. (2018). Fisetin Exerts Antioxidant and Neuroprotective Effects in Multiple Mutant hSOD1 Models of Amyotrophic Lateral Sclerosis by Activating ERK. Neuroscience 379, 152–166. 10.1016/j.neuroscience.2018.03.008 29559385

[B54] Watanabe S. Hayakawa T. Wakasugi K. Yamanaka K. (2014). Cystatin C Protects Neuronal Cells against Mutant Copper-Zinc Superoxide Dismutase-Mediated Toxicity. Cel Death Dis 5 (10), e1497. 10.1038/cddis.2014.459 PMC4237269 25356866

[B55] Wen D. Cui C. Duan W. Wang W. Wang Y. Liu Y. (2019). The Role of Insulin-like Growth Factor 1 in ALS Cell and Mouse Models: A Mitochondrial Protector. Brain Res. Bull. 144, 1–13. 10.1016/j.brainresbull.2018.09.015 30414993

[B56] Yamagata M. Yamamoto A. Kako E. Kaneko N. Matsubara K. Sakai K. (2013). Human Dental Pulp-Derived Stem Cells Protect against Hypoxic-Ischemic Brain Injury in Neonatal Mice. Stroke 44 (2), 551–554. 10.1161/STROKEAHA.112.676759 23238858

[B57] Yin X. Ren M. Jiang H. Cui S. Wang S. Jiang H. (2015). Downregulated AEG-1 Together with Inhibited PI3K/Akt Pathway Is Associated with Reduced Viability of Motor Neurons in an ALS Model. Mol. Cel. Neurosci. 68, 303–313. 10.1016/j.mcn.2015.08.009 26320681

[B58] Yoshino H. (2019). Edaravone for the Treatment of Amyotrophic Lateral Sclerosis. Expert Rev. Neurother 19, 185–193. 10.1080/14737175.2019.1581610 30810406

