# Supplementary material for: Stem Cells From Human Exfoliated Deciduous Teeth-Conditioned Medium (SHED-CM) is a Promising Treatment for Amyotrophic Lateral Sclerosis
Source: Front Pharmacol. 2022 Feb 3;13:805379. doi: 10.3389/fphar.2022.805379 (PMC8850386; doi:10.3389/fphar.2022.805379)
Supplement: Supplementary file 1 [file DataSheet3.DOCX]

**
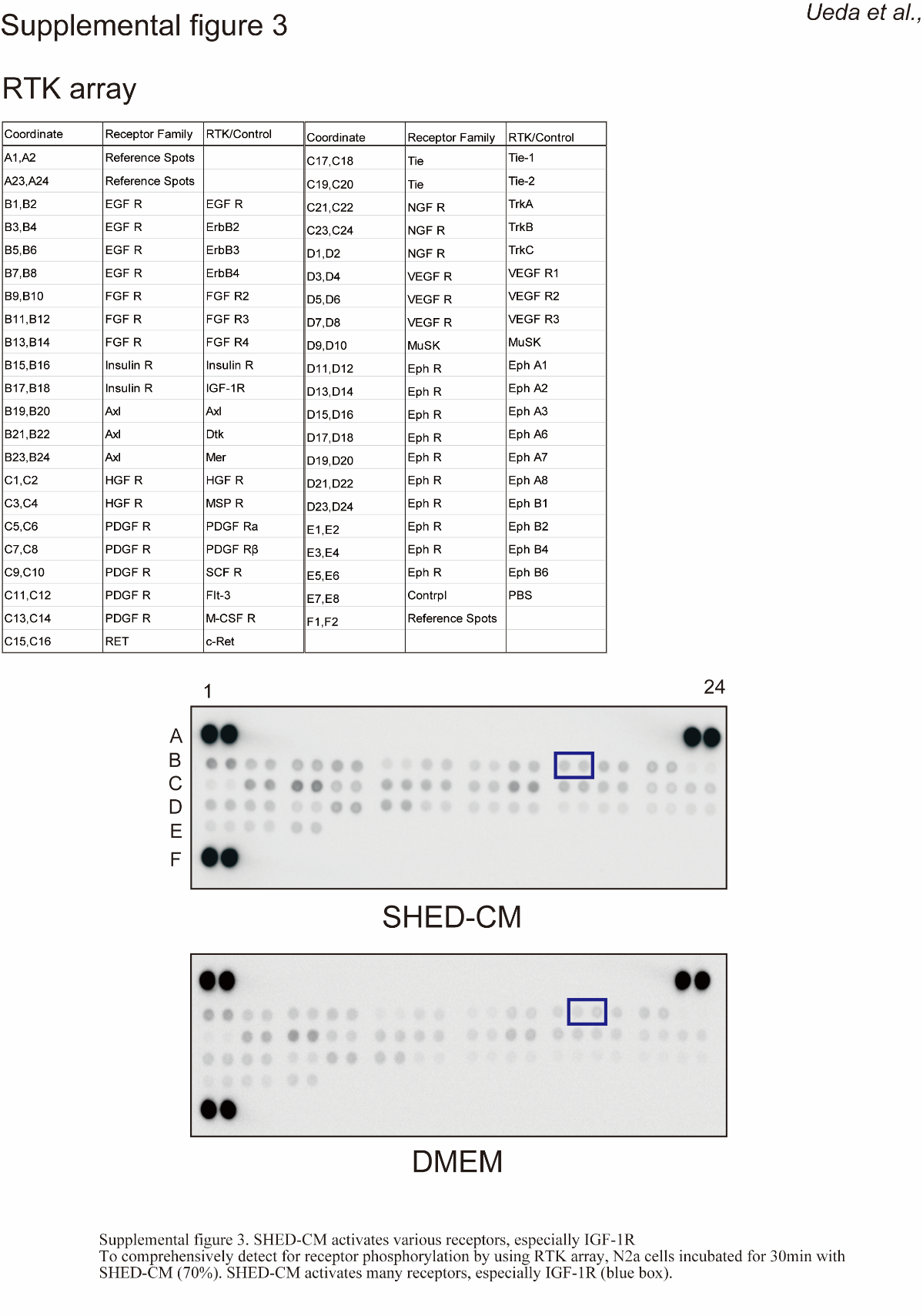
**

**Supplementary Figure 3.** **SHED-CM activates various receptors, especially IGF-1R**

To comprehensively detect for receptor phosphorylation by using RTK array, N2a cells incubated for 30min with SHED-CM (70%). SHED-CM activates many receptors, especially IGF-1R (blue box).
