# Supplementary material for: Stem Cells From Human Exfoliated Deciduous Teeth-Conditioned Medium (SHED-CM) is a Promising Treatment for Amyotrophic Lateral Sclerosis
Source: Front Pharmacol. 2022 Feb 3;13:805379. doi: 10.3389/fphar.2022.805379 (PMC8850386; doi:10.3389/fphar.2022.805379)
Supplement: Supplementary file 2 [file DataSheet2.DOCX]

**
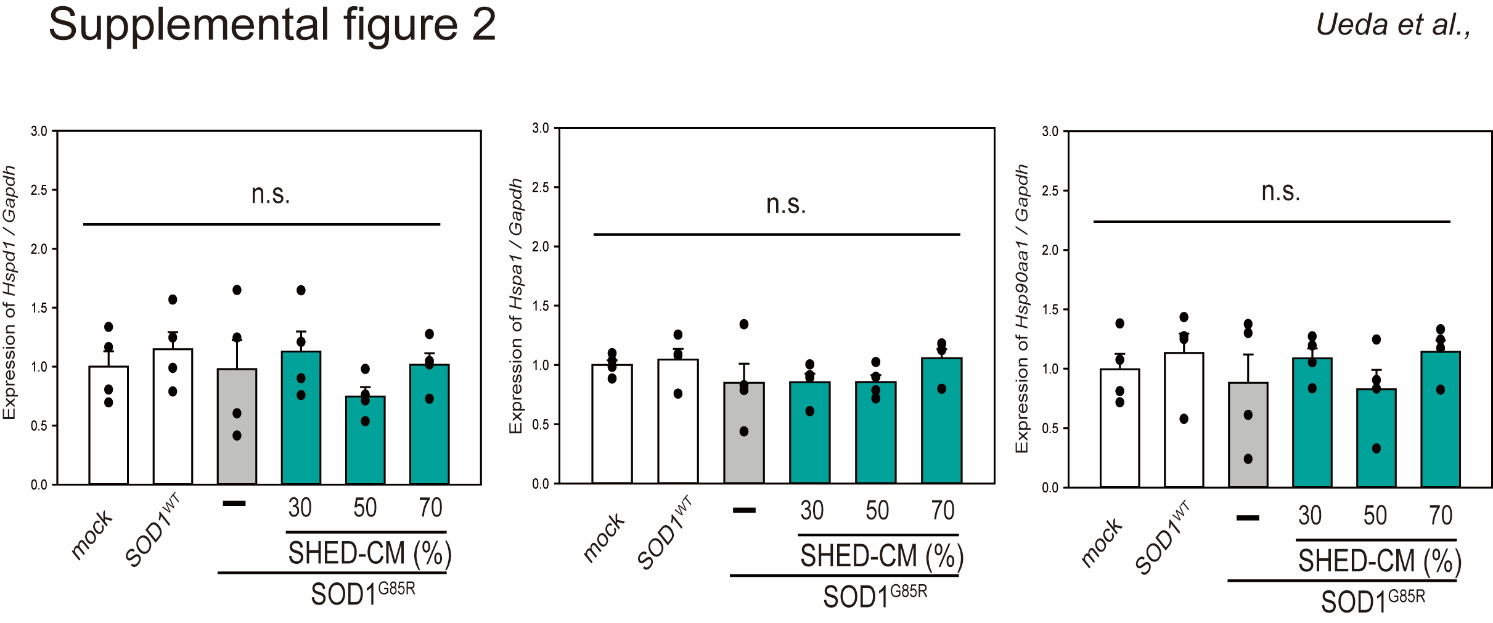
**

**Supplementary Figure 2.** **SHED-CM had no effect on the expression of HSP genes (*Hspd1, Hspa1, and Hsp90aa1*).**

N2a cells expressing mCherry-SOD1^G85R^ were treated with SHED-CM (30%, 50%, and 70%) for 24 h, HSP-related gene mRNA expression of *Hspd1, Hspa1, Hsp90aa1 and Gapdh* were analyzed using the SYBR Green-based RT-qPCR assay. The expression levels of mRNAs were normalized to the expression level of *Gapdh* mRNA. Results are presented as means ± SEM of three independent experiments based on the fluorescence intensity of the “mock” (mock = 1). n.s.: not significant.
