# Supplementary material for: Stem Cells From Human Exfoliated Deciduous Teeth-Conditioned Medium (SHED-CM) is a Promising Treatment for Amyotrophic Lateral Sclerosis
Source: Front Pharmacol. 2022 Feb 3;13:805379. doi: 10.3389/fphar.2022.805379 (PMC8850386; doi:10.3389/fphar.2022.805379)
Supplement: Supplementary file 3 [file DataSheet1.DOCX]

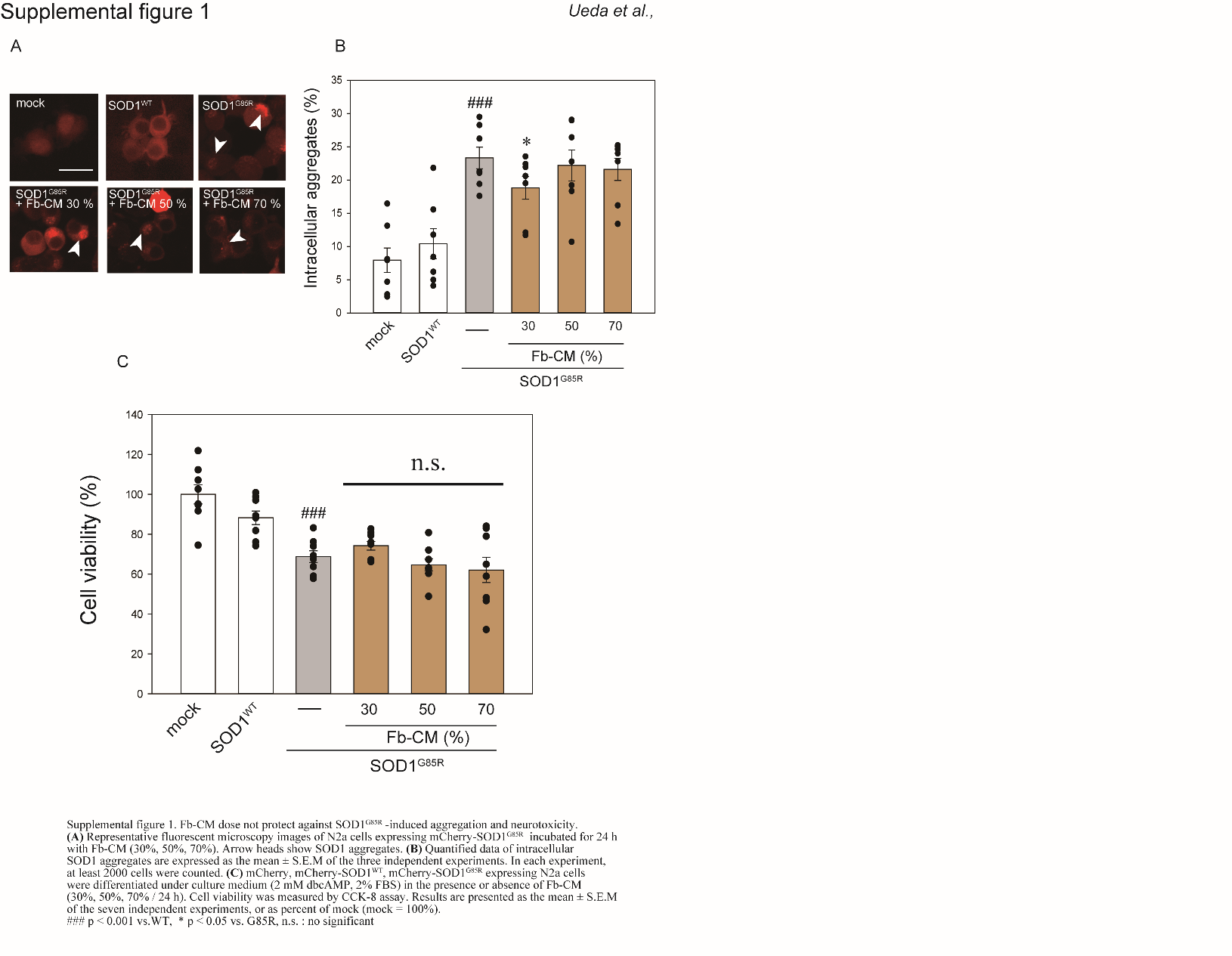


**Supplementary Figure 1.** **Fb-CM does not protect against SOD1G85R-induced aggregation and neurotoxicity.**

**(A):** Representative fluorescent microscopy images of N2a cells expressing mCherry-SOD1G85R-incubated for 24 h with Fb-CM (30%, 50%, 70%). Arrow heads show SOD1 aggregates. **(B):** Quantified data of intracellular SOD1 aggregates are expressed as the mean ± S.E.M of the three independent experiments. In each experiment, at least 2000 cells were counted. **(C):** mCherry, mCherry-SOD1WT, mCherry-SOD1G85R expressing N2a cells were differentiated under culture medium (2 mM dbcAMP, 2% FBS) in the presence or absence of Fb-CM (30%, 50%, 70% / 24 h). Cell viability was measured by CCK-8 assay. Results are presented as the mean ± S.E.M of the three independent experiments, or as percent of mock (mock = 100%). ### p < 0.001 vs.WT, * p < 0.05 vs. G85R, n.s. : no significant
